# Formulation of New Media from Dairy and Brewery Wastes for a Sustainable Production of DHA-Rich Oil by *Aurantiochytrium mangrovei*

**DOI:** 10.3390/md20010039

**Published:** 2021-12-29

**Authors:** Giovanni L. Russo, Antonio L. Langellotti, Vito Verardo, Beatriz Martín-García, Prospero Di Pierro, Angela Sorrentino, Marco Baselice, Maria Oliviero, Raffaele Sacchi, Paolo Masi

**Affiliations:** 1 Unit of Food Science and Technology, Department of Agricultural Sciences, University of Naples Federico II, 80055 Portici, Italy; prospero.dipierro@unina.it (P.D.P.); sacchi@unina.it (R.S.); masi@unina.it (P.M.); 2 CAISIAL Center, University of Naples Federico II, Via Università 133, 80055 Portici, Italy; langello@unina.it (A.L.L.); angela.sorrentino@unina.it (A.S.); marco.baselice@unina.it (M.B.); maria.oliviero2@unina.it (M.O.); 3 Department of Nutrition and Food Science, Campus of Cartuja, University of Granada, 18071 Granada, Spain; vitoverardo@ugr.es (V.V.); bea91mg@ugr.es (B.M.-G.); 4 Biomedical Research Center, Institute of Nutrition and Food Technology ‘José Mataix’, University of Granada, Avda del Conocimiento sn., 18100 Armilla, Spain

**Keywords:** microalgae, food waste, PUFA, DHA, bioconversion, sustainability, carotenoids

## Abstract

Mozzarella stretching water (MSW) is a dairy effluent generated from mozzarella cheese production that does not have a real use and is destined to disposal, causing environmental problems and representing a high disposal cost for dairy producers. Spent brewery yeast (SBY) is another promising food waste produced after brewery manufacturing that could be recycled in new biotechnological processes. *Aurantiochytrium mangrovei* is an aquatic protist known as producer of bioactive lipids such as omega 3 long chain polyunsaturated fatty acids (ω3 LC-PUFA), in particular docosahexaenoic acid (DHA). In this work MSW and SBY have been used to formulate new sustainable growth media for *A. mangrovei* cultivation and production of DHA in an attempt to valorize these effluents. MSW required an enzymatic hydrolysis to enhance the biomass production. The new media obtained from hydrolysed MSW was also optimized using response surface methodologies, obtaining 10.14 g L^−1^ of biomass in optimized medium, with a DHA content of 1.21 g L^−1^.

## 1. Introduction

Long chain ω-3 polyunsaturated fatty acids (LC-PUFA) have a series of beneficial effects on human health [1]. Among them, docosahexaenoic acid (DHA, C22:6n-3) is an important fatty acid, as it is one of the major components of the central nervous system [2]. Moreover, dietary DHA supplementation has been shown to be important in the prevention of cardiovascular diseases, schizophrenia, and specific types of cancer [3]. 

Actually, the principal source of DHA is fish oil, but it has several disadvantages, especially the low sustainability, the contamination by marine pollutants, fish allergy issues and an undesirable fishy smell [4,5]. Thraustochytrids, a heterotrophic fungus-like clade of Stramenopiles, represent a potential alternative to fish oil due to their high biomass and DHA productivity, which is much higher than the fish source [6]. Among the thraustochytrids, *Aurantiochytrium* (known as *Schizochytrium* until 2007) is a genus industrially exploited for the production of DHA [7]. *Aurantiochytrium* can produce high amounts of lipids (up to 55% of dry weight) and most of that is DHA (up to 35% of total fatty acids) [7]. For the industrial production of DHA, the price of growth medium represents a significant portion of the production costs, as well as the costs for preparing artificial sea water [8]. For that reason, new biotechnological processes based on the recycling of low cost side-streams from food industries would be an interesting way to produce omega-3 oil with lower production costs. The utilization of aquatic protists for treatment of food waste is gaining attention from the scientific community, thanks to their great metabolic flexibility and bioremediation capacities [9]. *Aurantiochytrium* sp. has been tested on different types of food waste, showing a high metabolic versatility to utilizing different type of organic and nitrogen sources [10,11].

The dairy industry is one of the main food industries in Italy and Europe, with tons of cheese produced every year. In Italy the main dairy product is the mozzarella cheese, with a production of more than 250,000 tons every year [12]. During the mozzarella manufacturing process, two main side-streams are generated: the cheese whey (CW) and the mozzarella stretching water (MSW). MSW is the effluent generated after the stretching step. It is treated as an effluent by the dairy companies because of its high salinity and high chemical oxygen demand (COD), and therefore it represents a serious environmental issue [13]. Nevertheless, dairy wastewaters are liquids rich in interesting compounds such as lactose (up to 5% *w/v*), proteins (up to 1% *w/v*), and other minor components (mineral salts, lactic acid and vitamins). Dairy by-products could be useful for the formulation of more sustainable microbial media [14], in particular for heterotrophic microorganisms that require high amounts of organic carbon and other nutrients for their growth.

Another promising food waste for biotechnological applications is the spent brewery yeast (SBY). It is an organic waste from brew manufacturing with a high content of proteins, free amino nitrogen (FAN), phosphates and other essential mineral salts [11]. These characteristics make this food waste very promising for microalgal cultivation [15]. In fact, SBY could be used in the formulation of a new medium obtained mainly from food wastes. 

To the best of our knowledge, *Aurantiochytrium* cultivation was never tried on dairy wastewater. We found only one paper about testing the saline wastewater from demineralization of CW for the cultivation of *Schizochytrium limacinum* PA-968 [8]. 

Therefore, the purpose of this study was to investigate the potential of a dairy wastewater (MSW) in combination with SBY as a new sustainable growth medium for *Aurantiochytrium mangrovei* cultivation. The chemical characteristics of MSW have been defined, and the new media optimized through response surface analysis with supplementation of nitrogen from brewery waste. The biochemical composition and lipid content of the obtained biomass have also been determined.

## 2. Results and Discussion

### 2.1. Characterization of MSW

The chemical and physical characterizations of reverse osmosis concentrated MSW (3:1) are reported in Table 1. 

The chemical composition of MSW is not well defined in scientific literature. This wastewater is interesting for the amount of reducing sugars (up to 23 g L^−1^), and also for a residual content of proteins (3.6 g L^−1^). The content of free amino nitrogen (FAN) is also relevant (0.174 mg L^−1^), as it is easily metabolized by aquatic protists, boosting their growth [16].

Moreover, the high amount of ash and chlorides make this waste a candidate for fermentation by marine microorganisms (i.e., *Aurantiochytrium mangrovei*). In fact, a high saline content is a common characteristic for this type of dairy effluent. The phosphorus (P) content reported was 87 mg L^−1^, which is a good amount for protist cultivation because it is an essential macronutrient for energy transfer and synthesis of phospholipids and nucleic acids [17]. Considering that the P content of standard YEP medium is estimated to be 70–90 mg L^−1^, the MSW alone can satisfy the P demand of *A. mangrovei*. Magnesium and calcium reported were high if compared to standard medium. However, this amount of Mg^2+^ could positively affect the cultivation, because magnesium ions act as a cofactor for malic enzyme, which converts malic acid to pyruvate during the transdehydrogenase cycle [18]. 

MSW showed a low pH value (3.55), probably due to the presence of citric and lactic acid. The latter is produced after natural microbial fermentations of the stretching water by lactic acid bacteria (LAB) or other species of the present microbiota [19]. The presence of organic acids could be interesting for heterotrophic or mixotrophic cultivation of microorganisms. These factors contribute to the high organic load that characterizes the dairy effluents; in fact, the COD found was more than 33 g L^−1^, which is economically critical for producers that need to dispose of this type of wastewater.

Comparing these results with other dairy wastewater characterizations, we found that MSW has a lower pH value (3.5 vs. 4.7–5.1), a higher content of total nitrogen (TN) (900 mg/L vs. 140–800 mg/L) and a lower amount of reducing sugars (23.1 g/L vs. 30–47 g/L) [20,21,22]. 

Nevertheless, the characteristics of MSW showed a promising nutrient composition for fermentations by marine protists. However, the amount of nitrogen and organic carbon are lower with respect to the standard YEP medium used for *A. mangrovei* cultivation.

### 2.2. Screening Tests Results

#### 2.2.1. Evaluation of Organic Carbon Sources 

**Figure 1:**
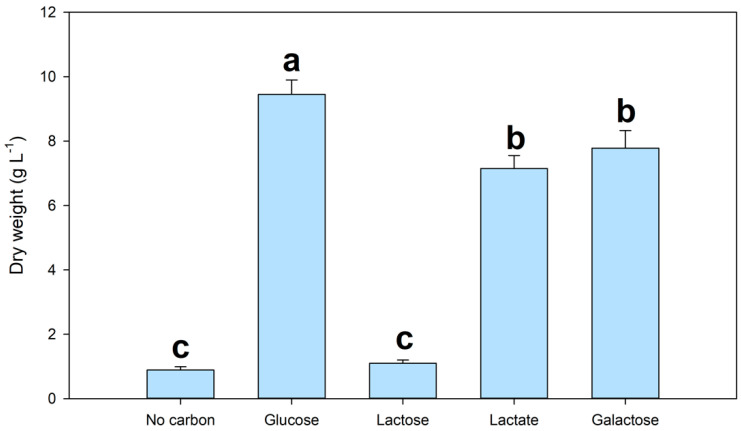
Screening test for different type of organic carbon source for *A. mangrovei* cultivation. The basal medium used for this test is yeast extract-peptone medium (YEP). Different letters mean a significant difference (*p* < 0.05) with *n* = 3.

The performance of *A. mangrovei* growth with different organic carbon sources is reported in Figure 1.

The protist showed a significant growth when using glucose, lactate and galactose as a source of organic carbon. This result is in line with other studies with the *Aurantiochytrium* species [10,23,24]. However, we found a significant difference (*p* < 0.05) between standard glucose, lactic acid and galactose. For lactose instead, no growth of *A. mangrovei* was observed after 72 h, proving the impossibility to metabolize this disaccharide. In fact, in another work where *Aurantiochytrium* sp. was tested on a media supplemented with demineralized CW, the thraustochytrid showed significant growth only when supplemented with glycerol [8]. Lactate has been tested as it is produced in the dairy wastewater following fermentations by the microbiota. This organic acid can affect positively the fermentation by *A. mangrovei*. A previous study evaluated the growth of *Schizochytrium* sp. using lactic acid instead of glucose [25], and the authors reported that the biomass growth with lactic acid medium was lower than the glucose medium. These findings are in line with our work. For galactose also, our results are in line with another work conducted with *Schizochytrium mangrovei* Sk-02 [24]. The authors reported the capability to metabolize this monosaccharide, but with less efficiency than glucose.

#### 2.2.2. Effect of MSW Hydrolysed and SBY on the Growth of *A. mangrovei*

**Figure 2:**
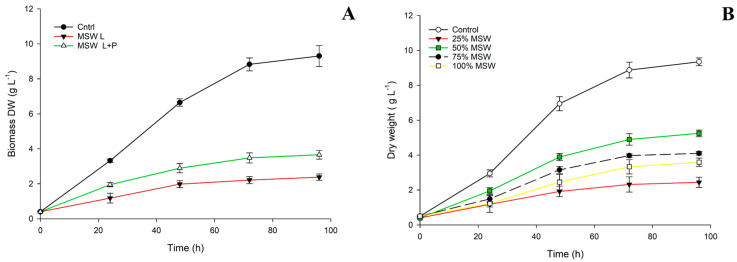
Evaluation of growth performance of *A. mangrovei* with MSW medium with only lactose hydrolysis (MSW L), with sequential lactose-protein hydrolysis (MSW L+P) (**A**) and screening of MSW hydrolyzed diluted at four different concentrations (25–100% *v/v*) (**B**). All the tests were conducted in triplicate (*n* = 3). The controls refer to YEP standard medium.

Since lactose is not metabolized by *A. mangrovei* (Figure 1) a hydrolysis of MSW was performed in order to increase the bioavailability of the nutrients present. The results of the first screening to evaluate the hydrolysis effect of MSW on *A. mangrovei* growth are reported in Figure 2A.

When using a medium in which only lactose was hydrolysed (MSW L), a lower biomass was obtained with respect to the samples’ growth with a medium subjected to sequential hydrolysis of proteins and lactose (MSW L+P). In fact, without the complete protein hydrolysis, a lower biomass growth can be observed in terms of dry cell weight (DW) (up to 2.2 g L^−1^) respect to the 3.63 g L^−1^ obtained on MSW L+P. After the sequential hydrolysis of MSW, the FAN content increased from 0.17 mg L^−1^ to 0.8 mg L^−1^, and this can explain the growth boost observed.

In fact, small peptides and FAN produced after proteolysis are used more efficiently by aquatic protists, enhancing their growth [9]. Also, the hydrolysis of lactose leads to glucose and galactose formation that can be easily metabolized by *A. mangrovei* (Figure 1a).

In the work of Pleissner et al. [16], a “fungal” hydrolysis with *A. awamori* and *A. oryzae* was used to enhance the nutrient availability of food waste. The authors tested the growth of *A. mangrovei* (called *Schizochytrium mangrovei*), reporting a higher biomass productivity respect to the control, thanks to FAN and glucose released after fungal pre-treatment.

However, the overall growth observed in our screening was very poor with respect to the standard medium. For that reason, we evaluated also the concentration effect of hydrolyzed MSW (lactose and protein hydrolysis) on the biomass production. In Figure 2B are reported the growth curves at different concentrations of MSW.

As we expected, when using this dairy effluent integrally without dilution (at 100% *v/v* of concentration), the growth performance was significantly lower than the control and 50% sample, obtaining only 2.1 g L^−1^ of DW after 96 h of cultivation. At 50% dilution instead, the growth was higher than the 100% and 75% samples, leading to 5.02 g L^−1^ of DW. This could be explained by a saline stress of the salts present in MSW or by other inhibitory substances present [26]. In fact, other authors reported a lower productivity when cultivating thraustochytrids at high salinity content [27,28]. Therefore, all the subsequent cultivation trials have been performed with hydrolyzed MSW diluted at 50% of concentration.

**Figure 3:**
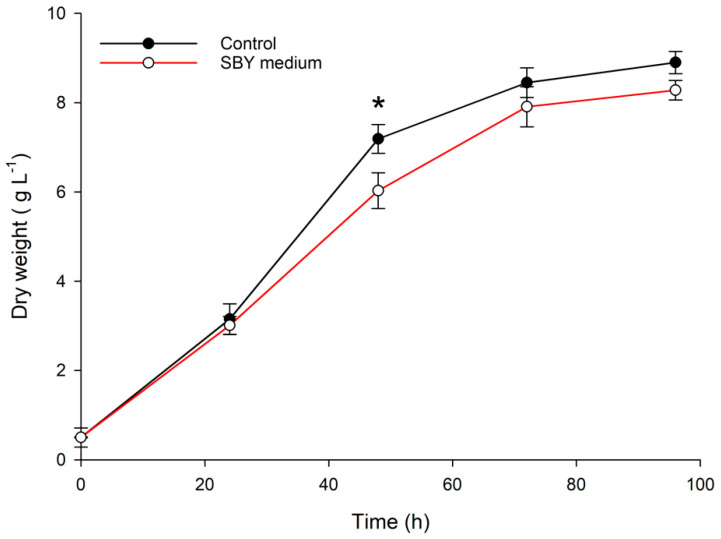
Growth curves of *A. mangrovei* cultivated with spent brewery yeast as the sole nitrogen source compared to standard medium. * means a significant difference (*p* < 0.05) with *n* = 3.

In our work, the ratio of the hydrolysis was the same, but the nutrients present in MSW were not sufficient to obtain a growth similar to the standard media (YEP medium). In fact, the nitrogen content of the standard medium was 0.95 g L^−1^ while the MSW diluted at 50% contains 0.46 g L^−1^ of N. For that reason, we tested a waste from the brewery industry (SBY) as an alternative nitrogen source that could satisfy the nutritional demand of the microalga. The SBY screening test results are reported in Figure 3.

Substituting the nitrogen of standard media (from yeast extract and peptone) with nitrogen from SBY, no significant difference after 72 h and 96 h of cultivation was observed. A significant difference (*p* = 0.04) can be observed only at 48 h of cultivation, likely due to a slower uptake of nitrogen from SBY with respect to the standard YE. With this result, SBY could be used to compensate the lack of nitrogen of MSW medium. 

This growth performance is in line with a previous work which obtained a biomass productivity (g L^−1^ day^−1^) of 3.52 ± 1.06 by *Aurantiochytrium* sp. KRS101 when using SBY extracted after autolysis with distilled water as the only nitrogen source [11].

### 2.3. Optimization of New MSW Hydrolysed Media

In order to increase the biomass productivity, MSW medium required a nutrient supplementation. CCD was used to examine the optimal supplementation of organic carbon (glucose) and nitrogen (SBY) in the new MSW media. The response surface design employed gave 13 combinations of selected nutrients (glucose and SBY) with three levels (−1, 0, +1). Table 2 reports the design and the results with the responses. Biomass concentration (expressed as g L^−1^ of DW) was used as response, and was calculated at log phase (72 h).

The significance of the model and its second-order Equation (2), derived from the multiple regression analysis of the data, was tested by analysis of variance (ANOVA) (Table 3) and a *p*-value lower than 0.05 was considered significant in the analysis.

The model fit is also expressed with coefficient of determination (R^2^), which was 0.9768, indicating that 97.68% of the variability in the Y (response) could be explained by the model. The *p*-value of the model was (*p* < 0.005), which implied that the model was significant; furthermore, the lack of fit is non-significant (*p* > 0.05), proving the validity of the model. Moreover, the predicted value observed from the model was not significantly different from the experimental value. The regression equation obtained from the model has been shown in Equation (1):

(1) $$\mathrm{Biomass}\;\mathrm{dry}\;\mathrm{weight}\;\left({\mathrm{gL}}^{-1}\right)=4.736+0.194\;\mathrm{Glu}+1.816\;\mathrm{SBY}-0.00384\;\mathrm{Glu}*\mathrm{Glu}-0.2433\;\mathrm{SBY}*\mathrm{SBY}+0.00067\;\mathrm{Glu}*\mathrm{SBY}$$

Based on ANOVA analysis, both factors showed significant impact on the growth of *A. mangrovei*. The most significant factor was glucose (*p* = 0.003) followed by SBY (*p* = 0.011).

**Figure 4:**
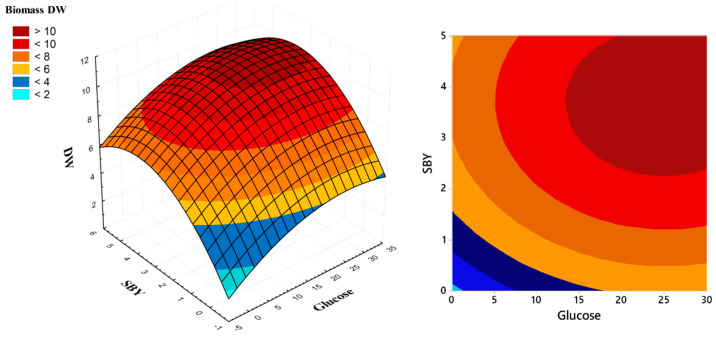
Surface (**left**) and contour plot (**right**) of RSM-CCD elaborated for *A. mangrovei* cultivated in MSW medium supplemented with spent brewery yeast and glucose.

In the run n. 13, without the addiction of glucose or SBY, the biomass obtained was 5.1 g L^−1^, while the highest DW value was obtained in run 2 (10.24 g L^−1^) with a combination of 2.5 g L^−1^ of SBY and 30 g L^−1^ of glucose. With supplementation of SBY and organic carbon, the biomass productivity was doubled. To better understand the RSM results, a 3-D surface plot and a contour plot were elaborated and reported in Figure 4.

From the figure it is possible to observe that a glucose supplementation higher than 15 g L^−1^ and 3.26 g L^−1^ of SBY are useless in terms of biomass productivity. In fact, the biomass DW seems to be stable at 10 g L^−1^ after these values. The optimal concentrations of factors extrapolated from regression equations are: 15.34 g L^−1^ of glucose and 3.22 g L^−1^ of SBY to supplement at MSW medium to obtain a biomass higher than 10 g L^−1^. 

For the optimal concentration of nitrogen, other works reported an optimal concentration of YE at 10–15 g L^−1^ for *Aurantiochytrium mangrovei* [29,30], while for this work it is 3.26 g L^−1^, suggesting the utilization of hydrolyzed proteins and FAN present in MSW by the microalga as N source. 

### 2.4. Model Confirmation and Characterization of Biomass Obtained with New MSW Optimized Medium

Once the optimal formulation of new MSW media was established, we confirmed the predicted biomass of the model cultivating *A. mangrovei* with a supplementation of 15.34 g L^−1^ of glucose and 3.22 g L^−1^ of SBY in MSW media. Moreover, we evaluated the lipid content and the nutrient consumption of the new medium. The results are reported in Table 4.

The biomass obtained was in line with the prediction of the CCD model. No significant differences were observed with the standard media in terms of biomass and lipid productivity. The FAN depletion of new MSW media was higher than the control, while the sugar consumption was very similar. This proves the optimal utilization of nutrients present in MSW media by *A. mangrovei*.

**Figure 5:**
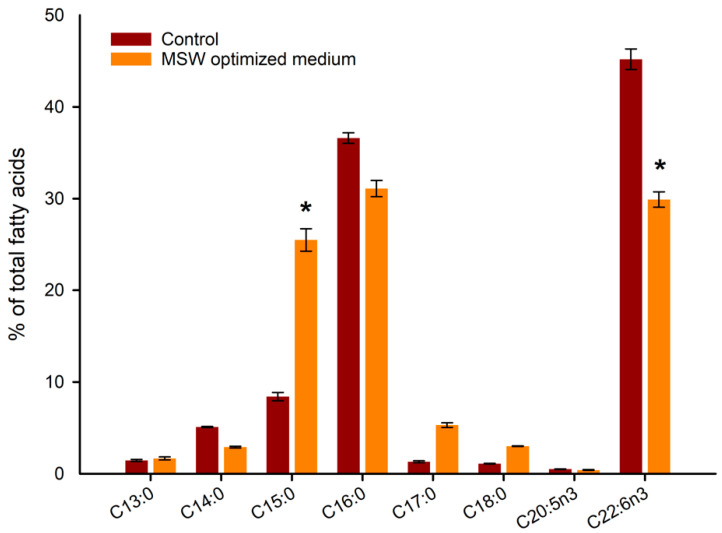
Fatty acids profile of extracted lipid from new MSW optimized media and standard media. (*) means a significant difference (*p* < 0.05) with the control.

To better understand the differences between the samples, the fatty acids profile was analyzed and reported in Figure 5.

Significant differences were observed in the fatty acids profile of MSW optimized media with the relative control in standard conditions. The DHA percentage of MSW medium was significantly lower than the control (30.6% vs. 45% respectively). This difference could be explained by the nutrient difference between the control and the new medium with different C/N ratios. It has been reported that fatty acids yields decrease when the carbon source is completely depleted, forcing the cells to consume their own reserves of lipids [31]. In fact, in the work of Wang et al. [32], 70 g/L of glucose were added to tofu whey wastewater to provide an extra carbon source in order to enhance lipid accumulation of *Schizochytrium* sp. S31. Supplementation of extra glucose to a food waste medium is also reported in another study with *Aurantiochytrium* sp. KRS101. In that work, the lipids and biomass productivity were enhanced after glucose supplementation [33]. In our case, the focus was not the optimization of DHA yield but the development of a new sustainable medium for *A. mangrovei* cultivation using dairy and brewery waste. Nevertheless, the DHA percentage registered with MSW media was higher than that reported in the literature of *Aurantiochytrium* grown on food waste [32] and in line with another medium based on orange peel extract [33] and on food waste hydrolysate [34].

The content of pentadecanoic acid (C15:0) is also worthy of mention because the percentage observed in MSW medium is significantly higher than the control. In fact, 25.5% of C15 has been observed in the new medium. Odd-carbon fatty acids have been used for anaplerotic therapy for Alzheimer’s disease, diabetes, cancer and cardiac disorder [35,36] and represents another high added value molecule. The concentration of C15 found in our study is higher than another work conducted on *Aurantiochytrium* sp. SA-96 [36], where the authors studied the influence of medium components on the production of C15. In fact, the authors found that adding soy milk to the culture medium increased the production of C:15. This could be a similar case as our study.

To further define the high added value compounds obtainable from *A. mangrovei* biomass, we evaluated the carotenoids from the biomass cultivated in standard condition, with MSW optimized medium and with MSW medium at higher luminosity (Table 5).

The control showed a low amount of carotenoids when compared to the other samples. Thraustochytrids are known to synthesize different carotenoids including β-carotene, astaxanthin, zeaxanthin, cantaxanthin, phoenicoxanthin and echinenone [37]. In our case, the only carotenoids found in *A. mangrovei* cultivated with standard medium are β-carotene and canthaxanthin. For the biomass obtained with MSW media instead, we found a higher content of β-carotene. This difference could be explained by the different composition of the medium, and by the presence of a different saline content that could stress the microorganism, stimulating the pigment production. Moreover, we cultivated *A. mangrovei* in MSW medium with an exposure of high luminosity during cultivation with a light intensity of 200 μmol photons m^−2^ s^−1^ in an attempt to enhance the pigment productivity. Astaxanthin and violaxanthin were detected in that condition, increasing the high added value of the biomass obtained with food waste. In fact, biosynthetic production of carotenoids and pigment from aquatic protists are influenced by several factors, such as light exposition, saline stress and nutrient composition [38]. In scientific literature the carotenoid profile of *Aurantiochytrium mangrovei* is not well described, however the carotenoids found were lower with respect to *Aurantiochytrium* sp. SK4 [39], but in line with *Aurantiochytrium limacinum* ATCC MYA-1381 [40].

### 2.5. Economic Considerations

The utilization of an organic substrate and a nitrogen source are one of the main costs of heterotrophic cultivation. The utilization of pure chemicals such as glucose and yeast extract are not economically feasible for large scale productions of thraustochytrids [41]. Moreover, in order to compete with DHA from fish oil, many companies are focusing on the research of low cost nutrients to use for their fermentation processes. Therefore, the nutrient recovery from food waste and by-product is an important step to achieve. The most expensive carbon source is glucose. In 2010 its cost in the international market reached $500/ton [42], accounting for 23–34% of total production cost for heterotrophic cultivations [33]. However, the main issue for nutrient cost of heterotrophic protists is the nitrogen source, which is more expensive than organic carbon. In fact, the cost of YE in 2010 was assessed to be around 9.2 $/Kg (9200 $/ton) [43]. 

A recent study reported that for production of DHA oil from *Schizochytrium* sp., it is possible to reduce the nutrient media cost of >70% using nitrogen from tofu wastewater [32]. In our case, the nitrogen source is completely replaced by the nutrients present in MSW and SBY, with only a small supplementation of glucose. Moreover, the glucose supplementation could be also replaced by other cheap organic carbon sources from food waste, as reported in our previous study [10].

With the process proposed in this study, the nutrient cost could be significantly cut. Also, the artificial seawater of the standard media has been completely replaced by MSW media, which results in a reduction in the cost of artificial seawater and mineral salts. Further studies are required to understand the economic benefit of using these food processing by-products in substitution of the standard medium, most of all a techno-economic assessment of the whole process.

## 3. Materials and Methods

### 3.1. Food Waste Samples and Chemical Characterization

MSW samples were gently provided by a mozzarella cheese factory (*Capurso Azienda Casearia* srl, Gioia del Colle, Bari, Italy) which concentrates the MSW using reverse osmosis in order to reduce the volume to be sent to wastewater treatment plant.

The concentrated MSW samples were taken from the accumulation tanks of the factory, aliquoted and immediately frozen at −20 °C to prevent any fermentation. Prior any analysis, the samples were filtered to remove big solid particulates that could interfere to the biomass growth. Pre-treatment of MSW consisted in a first neutralization from pH 3.5 to 7.0 using NaOH 5 M. After that, the samples were heated to 80 °C for 10 min and then centrifuged at 14,000× *g* for 7 min to remove the precipitate [8]. The supernatant was collected and sterilized at 121 °C for 15 min.

Spent brewery yeast (SBY) was obtained from an artisanal brewery factory. SBY was aliquoted and frozen at −20 °C. Hydrolysis of SBY was obtained by the standard autolysis method reported by Jacob et al. [44]. Autolysis in the distilled water reported was best in terms of cell growth and economic feasibility. After autolysis, the DW of SBY lysate was 47.5 g L^−1^ and TN was 4.51 g L^−1^.

### 3.2. Organism and Cultivation

*Aurantiochytrium mangrovei* (RCC893) was obtained from the Roscoff algae collection (Roscoff, France). A stock culture of an axenic microalga strain was maintained routinely by regular sub-culturing at 2-week intervals on both liquid and agar slants of YEP Medium obtained from half-strength artificial seawater (17.5 g L^−1^ of sea salts) with 30 g L^−1^ of glucose, adjusted at pH 6.5. The nitrogen (N) source was peptone (2 g L^−1^) and yeast extract (5 g L^−1^). The algae were cultivated in the presence of light (light intensity, 50 μmol photons m^−2^ s^−1^) at temperature of 25 ± 2 °C. Culture agitation was provided by means of an orbital shaker at 200 rpm.

### 3.3. Experimental Design

The experimental design consisted of four steps: (1) a screening test to evaluate the behavior of the protist in the presence of various carbon sources and with various enzymatic hydrolysis of MSW; (2) evaluation of different concentrations of MSW as basal medium and screening for SBY as the only nitrogen source; (3) a central composite design (CCD) for the determination of the optimal supplementation of SBY and glucose to MSW medium; and (4) characterization of the biomass obtained in terms of high added value products (lipids and carotenoids).

#### Screening Tests

For the screening test, three trials were conducted: the first for the evaluation of *A. mangrovei* growth under different types of carbon source; the second to evaluate the growth of *A. mangrovei* in the presence of different concentrations of MSW; the third to evaluate the utilization of SBY as main nitrogen source for *A. mangrovei*.

For the first screening, the carbon sources used were glucose, galactose, lactose and lactic acid, adding the same amount of carbon (in g L^−1^) of the control with glucose. The second screening was conducted using four concentrations of MSW: 25, 50, 75 and 100% (dilution *v/v*). The dilution was obtained using distilled water without the addition of any other nutrient. The third screening was made using SBY as the only nitrogen source in substitution of standard yeast extract. The salinity of all samples was set at 1.75% (*v/v*) using commercial Aquaforest® sea salts. A working volume of 300 mL was placed in a 500 mL Erlenmeyer flask for each concentration. *A. mangrovei* was inoculated into each flask to reach an initial concentration of 400 mg L^−1^ of DW. The experiment was carried out at 28 °C and the mixing was provided through an air bubbling system equipped with a filter of 0.22 µm in order to prevent any contamination and to provide oxygenation to the culture.

### 3.4. Hydrolysis of Dairy Wastewater

Enzymatic hydrolyses of MSW were carried out using the method proposed by Bikash et al. [45] with minor modifications. A sequential hydrolysis has been conducted in order to obtain a hydrolysate without lactose and high molecular weight proteins. The protocol used was the following: 300 mL of MSW was heated at 85 °C for 1 h in a water bath in order to stabilize the product. After that, the samples were transferred on an orbital shaker set at 37 °C and a food grade lactase was added to the bottles (186 mg L^−1^). At the end, the samples were heated at 90 °C for 5 min to inactivate the lactase.

After this first step of hydrolysis, we began the proteolysis phase. The bottles were placed on an orbital shaker at 50 °C, 150 rpm and 12.5 mL of protease from *Aspergillus oryzae* (Merck, Rome, Italy) were added, corresponding to about 16,000 LAPU aminopeptidase units per liter of MSW. Proteolysis was carried out for 3 h. After this period, the enzyme was inactivated at 85 °C for 3 h. The samples obtained were frozen to prevent any fermentation.

### 3.5. Response Surface Analysis and Formulation of Optimized Media

The new media obtained from hydrolysed MSW was optimized using response surface methodology (RSM). RSM is one of the most effective method for the optimization of the fermentation process [46]. This method was applied to formulate the optimal combination of glucose (carbon content) and spent brewery yeast (nitrogen source) to supplement the new hydrolysed MSW medium in order to enhance the biomass production. SBY supplementation was expressed as g L^−1^ of lysate DW.

The RSM has been done by constructing a three level full factorial central composite design (CCD). The optimization consisted of 13 runs conducted in two blocks with 4 cubic points (or factorial points), 4 axial points (or star points) and 3 center points for each block. 

The mathematical relationship of the response (Y) to the significant independent variables X_1_ and X_2_ is given by the following quadratic polynomial Equation (2):

(2) $$Y\;={\;\beta }_{0}+\sum_{i=1}^{n}\beta_{i}X_{i}+\sum_{i=1}^{n}\beta_{\mathrm{ii}}X_{i}^{2}+\sum_{i=1}^{n}\beta_{\mathrm{ij}}X_{i}X_{j}$$

where Y is the predicted response; X_i_ and X_j_ are the coded values; β_0_ the independent coefficient; β_i,j_ is the linear coefficient associated to each independent factor (X_i,j_) and β_ij_ and β_ii_ are the coefficient for interaction and quadratic effects, respectively [47].

The optimal condition extrapolated from the model was also confirmed by cultivating *A. mangrovei* with the new nutrients parameters, and compared with the prediction of the model.

### 3.6. Analytical Methods

#### 3.6.1. Measurement of Dry Cell Weight

Every 24 h, 10 mL of culture volume were taken and transferred in weighted dry tubes, then centrifuged at 5000× *g* for 10 min. The supernatants were discarded, the pellets were washed twice with phosphate buffered saline (PBS) and dried overnight in an oven at 105 °C to obtain the dry cell weight (DW) [11].

#### 3.6.2. Chemical Characterization of Food Waste

For the evaluation of the chemical-physical composition, a different type of analysis has been performed using standard methods to obtain dry weight, ash, salt content, moisture, pH and protein content [17]. The ash content was determined gravimetrically until reaching a constant weight in a muffle furnace at 550 °C. The protein content was evaluated by the Bradford assay [48] using bovine serum albumin (BSA) as the standard (MilliporeSigma, Burlington, MA, USA) and a Shimadzu UV-1700 spectrophotometer (Kyoto, Japan) for the reading of the absorbance.

The determination of reducing sugars was obtained with the dinitrosalicylic assay (DNS) [49], and the lactose has been determined spectrophotometrically following the AOAC method 2006.06 [50]. The pH value was detected using a pH meter (Mettler Toledo, Switzerland). The salinity content was evaluated with a hand refractometer. The Mg^2+^, Cl^−^ and Ca^2+^ content were established following ISO standards [51]. FAN content was estimated with the ninhydrin reaction method described by [52].

#### 3.6.3. Lipid Extraction and Fatty Acid Methyl Esters (FAMEs)

The total amount of lipids were extracted according to a method previously established by Cha et al. [53], with minor modifications. 0.1 g of a powdered microalga sample was extracted with 3.33 mL of concentrated HCl (37%). The mixture was shaken using a vortex for 2 min and boiled twice at 100 °C for 20 min to induce cell disruption. The tubes were cooled at room temperature. Lastly, the lipid fraction was extracted three times: once with 4 mL of hexane and twice with 2.5 mL of hexane. The fatty acid methyl esters (FAMEs) were prepared from the total amount of previously obtained lipids by a transmethylation reaction utilizing a methodology previously described with certain modifications [54]. 20 mg of lipid extract was mixed with 50 µL 2N KOH in methanol, 500 µL of n-hexane and 500 µL of methylnonadecanoate (Sigma, St. Louis, MO, USA) as internal standard (1 mg/mL). The mixture was vortexed for two min. The upper layer supernatant (FAME extract) was collected and injected into a gas chromatography-mass spectrometer (GC-MS). Microalgal extracts were analyzed according to the method conditions previously described by Conde et al. [55]. The analyses were carried out by using an Agilent 7890A gas chromatograph coupled to a Waters QUATTRO microTM mass spectrometer detector. The separation was achieved on a capillary column DB-5MS (30 m × 0.25 mm; f.t. 0.25 µm) purchased from Agilent Technologies (J&W Scientific, Folsom, CA, USA). The oven temperature was 58 °C for 2 min, 25 °C min^−1^ to 160 °C, 2 °C min^−1^ to 210 °C, 30 °C min^−1^ to 225 °C (held for 20 min). The MS detector operates with an ionization energy of 70 eV and a scanning range of *m/z* 50–550 *m/z*. The conditions were helium as carrier gas at 1.4 mL min^−1^, the inlet temperature was 220 °C, the detector temperature was 230 °C, 2 µL of injection volume (splitless). Data were analyzed using MassLynx version 4.1 (Waters, San Jose, CA, USA).

#### 3.6.4. Determination of Carotenoids in Microalgal Extracts by HPLC/MS Analysis

The extraction was obtained by using an ultrasonic bath (Bandelin, Sonorex, RK52, Berlin, Germany), which operates at a frequency of 35 kHz according to the protocol described previously by Castro-Puyana et al. 2013 [56] with some modifications. Briefly, 10 mg of a sample of microalgal was added to 1.5 mL of ethanol containing 0.1% (*w*/*v*) of butylated hydroxytoluene. The mixture was centrifuged for 10 min at 10,000× *g* rpm (4 °C). The extracts were collected and filtered through 0.2 μm nylon syringe filters and stored at −18 °C until the analyses.

Microalgal extracts were analyzed by UPLC Acquity coupled XEVO-TQ-S Triple quadrupole mass spectrometry (Waters Corporation, Milford, MA, USA). Carotenoids were separated on an YMC-C30 reversed-phase column (250 × 4.6 mm. 3 µm). The mobile phases consisted of methanol with 5% water and 0.1% formic acid as mobile phase A and methyl tert-butyl ether as mobile phase B. The conditions of the solvent gradient were 60% A to 0% A in 30 min with a flow rate of 1 mL min^−1^. Analysis parameters were arranged using a positive-ion mode. The parameters of multiple reaction monitoring MRM transitions for all the standards are listed in supplementary material (Table S1). Additional mass spectrometric parameters were as follows: Source temperature was 150 °C, the desolvation temperature was 500 °C, cone gas flow 150 °C, the source offset was 30 V, the desolvation gas flow was 1000 L/h, the collision gas flow was 0.15 mL min^−1^, and the collision gas was argon. The data was acquired using MassLynx version 4.1 (Waters, San Jose, CA, USA). Carotenoids were quantified by standards of violaxanthin, astaxanthin, canthaxanthin and β-carotene. The calibration curves were prepared from the limit of quantification (LOQ) to 500–625 mg L^−1^. All calibration curves revealed a good linearity among different concentrations, and the determination coefficients were higher than 0.9918 in all cases. The method used for analysis showed a limit of detection (LOD) within the range 0.02–2.06 µg L^−1^ and the LOQ was within 0.08–6.85 µg L^−1^ (Table S2).

### 3.7. Statistical Analysis

All the analyses were carried out in triplicate, and average values with standard deviation were reported. One-way ANOVA was applied using raw data to test for significant differences among the samples (significance level was always set at *p* < 0.05). The Tukey’s test was used for post-hoc analysis when there were significant differences among the samples. The data were analyzed using IBM© SPSS© Statistics software Ver. 23 (SPSS, Inc., Chicago, IL, USA). RSM analysis was carried out using Statistica 7.0 package (StatSoft, Tulsa, OK, USA).

## 4. Conclusions

The combination of wastewater from mozzarella manufacturing and brewery waste showed a promising alternative for a more sustainable *Aurantiochytrium mangrovei* cultivation. Pre-treatment of MSW is mandatory to achieve an optimal biomass concentration and lipid production. Enzymatic hydrolyses achieved good growth performances in terms of biomass produced. However, a supplementation of nitrogen from spent brewery yeast and glucose is required to boost the growth of *A. mangrovei* using MSW.

The optimization with RSM leads to a biomass DW of 10.14 g L^−1^ with 38.9% of lipids and 29.8% of DHA on total FAME. The results are comparable to the relative growth with standard media. These findings suggest that hydrolyzed MSW with SBY can be used in new biotechnological processes in order to reduce nutrient costs for production of biomass that is rich in DHA oil.

## Figures and Tables

**Table 1:** Chemical and physical characterization of mozzarella stretching water.

Parameters	Value
pH	3.55 ± 0.2
Ash (g L^−1^)	26.2 ± 0.9
Dry weight (g L^−1^)	68.5 ± 1.1
N total (g L^−1^)	0.91 ± 0.09
Protein content (g L^−1^)	3.6 ± 0.2
Lactic acid (g L^−1^)	6.08 ± 0.79
Citric acid (g L^−1^)	1.03 ± 0.19
Free amino nitrogen (mg L^−1^)	0.174 ± 0.03
Reducing sugars (g L^−1^)	23.26 ± 0.4
Lactose (g L^−1^)	22.48 ± 0.7
Total sugars (g L^−1^)	24.12 ± 0.6
COD (mg L^−1^)	33506 ± 21.1
Cl^-^ (g L^−1^)	14.61 ± 1
Ca^2+^ (g L^−1^)	0.69 ± 0.05
Total P (mg L^−1^)	87.3 ± 1.25
Na^2+^ (g L^−1^)	7.66 ± 0.8
Mg^2+^ (mg L^−1^)	95.76 ± 3.7

Values expressed as mean (*n* = 3) ± SD.

**Table 2:** Experiment design and results of biomass growth optimization with supplementation of glucose and SBY by central composite design.

Run	Factor Assignment		Biomass Dry Weight (Y)	
	X_1_ (Glucose)	X_2_ (SBY)	Experimental Value (g L^−1^)	Predicted Value (g L^−1^)
1	−1	0	7.28	7.75
2	+1	0	10.24	10.06
3	+1	+1	9.79	9.99
4	0	+1	9.87	9.73
5	0	0	9.81	9.77
6	0	0	9.72	9.77
7	+1	−1	7.13	7.09
8	−1	+1	7.85	7.73
9	0	0	9.97	9.77
10	0	−1	6.38	6.78
11	0	0	9.57	9.77
12	0	0	10.14	9.77
13	−1	−1	5.10	4.73

Coded values: X_1 =_ glucose (g/L); X_2_ = SBY (g/L); the three levels (−1, 0 and +1) set for glucose were 0, 15 and 30 g L^−1^, while for SBY was 0, 2.5 and 5 g L^−1^ respectively.

**Table 3:** Analysis of variance for biomass production using coded values and regression equation.

Source	DF ^a^	Adj SS ^b^	Adj MS ^c^	F-Value	*p*-Value
Model	5	16.45	3.29	23.35	0.003
Glucose (X_1_)	1	4.59	4.59	32.60	0.005
SBY (X_2_)	1	1.27	1.27	9.07	0.011
Linear	2	5.87	2.93	20.84	0.001
Square	2	10.52	5.26	37.35	0.000
X_1_ * X_1_	1	3.22	3.22	22.91	0.004
X_2_ * X_2_	1	3.28	3.28	23.34	0.002
X_1_ * X_2_	1	0.05	0.05	0.36	0.868
Error	7	0.98	0.14		
Lack of Fit	3	0.87	0.29	9.19	0.129
Pure Error	4	0.11	0.02		
Total	12	17.4376			

R^2^ = 97.68 (^a^ DF, degree of freedom; ^b^ SS, sum of squares; ^c^ MS, mean squares; F, probability of distribution; *p*, probability).

**Table 4:** Comparison of biomass dry weight, lipid content, FAN and sugar consumption of *A. mangrovei* between standard media and new MSW medium optimized through CCD.

Parameter	Control	MSW Optimized Media
Biomass DW (g L^−1^)	9.44 ± 0.12	10.07 ± 0.23
Biomass productivity (g L^−1^ day^−1^)	3.14 ± 0.06	3.35 ± 0.08
Total lipids (%DW)	41.1 ± 1.2	38.9 ± 0.88
FAN consumption (%)	80.06	87.24
Sugar consumption (%)	92.61	94.59

All values are expressed an mean (*n* = 3) ± SD.

**Table 5:** Table of quantification of carotenoid in *A. mangrovei* by HPLC-MS. (Data are given as µg/g DW).

Sample	β-Carotene	Canthaxanthin	Astaxanthin	Violaxanthin
Control	0.34 ± 0.06 ^a^	0.62 ± 0.05 ^b^	Trace	n.d.
MSW media	2.93 ± 0.05 ^b^	0.29 ± 0.04 ^a^	Trace	n.d.
MSW media + light	1.85 ± 0.02 ^c^	0.27 ± 0.01 ^a^	0.28 ± 0.01	3.23 ± 0.08

Values are means ± SD (*n* = 3); n.d. = not detected.

## Data Availability

Not applicable.

## Supplementary Materials

The following supporting information can be downloaded at https://www.mdpi.com/article/10.3390/md20010039/s1, Table S1: Parameters for The MRM transitions, Table S2: Calibration curves of standards for the determination of carotenoid in microalgae.
