# Supplementary material for: Formulation of New Media from Dairy and Brewery Wastes for a Sustainable Production of DHA-Rich Oil by Aurantiochytrium mangrovei
Source: Mar Drugs. 2021 Dec 29;20(1):39. doi: 10.3390/md20010039 (PMC8778784; doi:10.3390/md20010039)
Supplement: Supplementary file 1 [file marinedrugs-20-00039-s001.zip › marinedrugs-1511606-supplementary.pdf]

Table S1. Parameters for The MRM transitions

Compound	Name	Parent	Cone voltage (V)	Daughters	Collision energy (V)
1	Fucoxanthin	641.41	4	109.03; 149.08; 119.05; 105.00	16; 18; 46; 48
2	Violaxanthin	601.47	52	221.11; 105.06; 119.04; 583.47	14; 46; 42; 4
3	Neoxanthin	600.58	12	105.06; 159.55; 142.58; 119.04	40; 30; 38; 34
4	Astaxanthin	597.48	10	147.07; 119.05; 173.08; 107.02	12; 46; 12; 36
5	Antheraxanthin	584.56	10	105.01; 119.05; 156.64; 145.06	44; 32; 32; 32
6	Meso-zeaxanthin	568.8	14	476.41; 104.45; 118.59; 144.68	8; 56; 34; 26
7	Zeaxanthin	568.52	10	476.38; 119.04; 105.00; 91.02	8; 34; 56;76
8	Lutein	568.5	8	476.36; 119.05; 105.01; 338.24	8; 34; 44; 10
9	Canthaxanthin	565.53	14	133.02; 203.11; 105.00; 363.25	32; 12; 58; 6
10	Echinenone	550.73	18	458.40 202.83 170.76 156.65	8; 14; 26; 30
11	β-Carotene	536.8	16	444.45 104.52 118.58 90.38	10; 48; 40; 60

Table S2. Calibration curves of standards for the determination of carotenoid in microalgae.

Compound	Name	LOD (ppb)	LOQ (ppb)	Calibration ranges (ppb)	Calibration curves (ppb)	R^2
1	Fucoxanthin	2.06	6.85	LOQ-500	58.3645x + 71.7837	0.9971
2	Violaxanthin	1.19	3.96	LOQ-625	100.941x + 574.666	0.9984
3	Neoxanthin	0.38	1.27	LOQ-625	314.358x + 1231.63	0.9964
4	Astaxanthin	0.40	1.33	LOQ-500	301.726x - 291728	0.9751
5	Antheraxanthin	0.51	1.70	LOQ-625	234.862x + 350.891	0.9962
6	Meso-zeaxanthin	0.02	0.08	LOQ-500	4861.13x + 4495.87	0.9958
7	Zeaxanthin	0.03	0.09	LOQ-625	4443.93x + 5109.34	0.9970
8	Lutein	0.12	0.41	LOQ-500	964.214x + 658.069	0.9958
9	Canthaxanthin	0.27	0.90	LOQ-500	443.201x - 150.188	0.9918
10	Echinenone	0.08	0.27	LOQ-625	1467.21x + 2007.5	0.9938
11	*β*-Carotene	0.02	0.08	LOQ-535	5254.84x + 2749.83	0.9976
